# Is anti-Mullerian hormone a good diagnostic marker for adolescent and young adult patients with Polycystic ovary syndrome?

**DOI:** 10.4274/tjod.21549

**Published:** 2015-12-15

**Authors:** Aytekin Tokmak, Hakan Timur, Rıfat Taner Aksoy, Mehmet Çınar, Nafiye Yılmaz

**Affiliations:** 1 Zekai Tahir Burak Women’s Health Education and Research Hospital, Clinic of Obstetrics and Gynecology, Ankara, Turkey

**Keywords:** anti-Müllerian hormone, adolescent, Polycystic ovary syndrome

## Abstract

**Objective:**

To evaluate serum anti-Mullerian hormone (AMH) levels in adolescent and young adult (AYA) Turkish patients with Polycystic ovary syndrome (PCOS), and to determine whether it had a diagnostic value.

**Materials and Methods:**

A total of 90 AYA patients were recruited for this study. The study group consisted of 43 patients diagnosed as having PCOS, and the control group comprised 47 age-matched patients. The diagnosis of PCOS was made in accordance with the recent Amsterdam European Society of Human Reproduction and Embryology/American Society for Reproductive Medicine PCOS consensus workshop group’s proposal that all three of the Rotterdam criteria for diagnosing PCOS in adolescents be present. In all patients, serum AMH levels were measured using enzyme-linked immunosorbent assay. Receiver operator characteristics (ROC) curve analysis was performed to reveal diagnostic potential of AMH.

**Results:**

Serum AMH levels were higher in the PCOS group compared with controls, but the difference was not statistically significant (10.1±6.9 ng/mL vs. 9.4±5.5 ng/mL, p=0.198). There was a significant age-related decrease in AMH levels in both the study and control groups (r=-0.331, p=0.001). There was also a significant inverse correlation between serum AMH and follicle-stimulating hormone levels in all patients (r=-0.227, p=0.031). ROC analyses demonstrated that the area under the curve indicative of AMH value for discriminating PCOS was 0.579 with a 95% confidence interval of 0.453-0.705 (p=0.198). The cut-off value according to the highest Youden index was calculated to be 14.0 ng/mL with a sensitivity of 48.8% and specificity of 77.1%.

**Conclusion:**

Serum AMH levels are slightly higher in AYA patients with PCOS than in controls. However, AMH is not a good marker for the diagnosis of PCOS in AYA patients.

## PRECIS:

AMH is not a good diagnostic marker for PCOS in adult and young Turkish patients.

## INTRODUCTION

Polycystic ovary syndrome (PCOS) is the most common endocrinologic disorder of reproductive-age women with a prevalence of 5 to 8%^(1)^. Obesity, dyslipidemia, type 2 diabetes mellitus, and impaired glucose tolerance are clinical features with a high prevalence in patients with PCOS. Women with PCOS run the risk of future cardiovascular disease associated with endothelial dysfunction and metabolic syndrome^(2)^. Therefore, considering the long-term consequences of the disease, early diagnosis and treatment of this syndrome is important to avoid these adverse effects.

PCOS has an ongoing clinical spectrum that starts from the early prepubertal years and continues after menopause. Misdiagnosis of disease may lead to unnecessary anxiety in patients and their families, whereas accurate diagnosis is important to prevent delayed treatment. Although a number of genetic and environmental factors are reported in the development of PCOS, the exact underlying pathophysiologic mechanism remains unclear^(3)^. The diagnosis of adolescent PCOS is controversial and challenging because of the conflict with the physiologic signs of puberty and heterogeneity in phenotypic manifestations. The criteria used for adults may not be validated for adolescents.

There are some historical consensuses on PCOS diagnosis. According to the latest of them, which was conducted by the Amsterdam European Society of Human Reproduction and Embryology the American Society for Reproductive Medicine (ESHRE/ASRM)-sponsored 3rd PCOS consensus group in 2012, the presence of all three of the Rotterdam criteria for the diagnosis of PCOS in adolescents is required^(4)^. Likewise, Sultan and Paris^(5)^ previously suggested that four of the following five criteria be present for the diagnosis of adolescent PCOS: clinical and biologic evidence of hyperandrogenism, hyperinsulinism, menarch age >2 years, oligo-/amenorrhea, and polycystic ovaries on ultrasonography. Although biochemical hyperandrogenemia is one of the laboratory markers for the diagnosis of PCOS, the validity of androgenic markers and their derived indices in the diagnosis is uncertain. The definition of clinical and biochemical hyperandrogenism in adolescents is also unclear. Therefore, a stand-alone diagnostic test that could be used accurately is needed.

Anti-Mullerian hormone (AMH), also known as Mullerian inhibiting factor, is a protein that belongs to the transforming growth factor-beta (TGF-β) family, which plays an important role in male sex differentiation by causing irreversible regression of the Mullerian ducts. In females, AMH has been proposed as a marker of ovarian aging and reserve^(6)^. It is only secreted by the granulosa cells in the preantral and antral follicles in the ovaries. Its highest concentrations are in small antral follicles and is very low or undetectable in follicles >10 mm. AMH plays a role in regulating ovarian activity. Additionally, it inhibits initiation of the development of primordial follicles and the selection of a high number of follicles by reducing follicle sensitivity to follicle-stimulating hormone (FSH)^(7^).

There is accumulating data suggesting that AMH could present as a biochemical marker for PCOS. However, most data about AMH and PCOS are from western countries, and on infertile adults. In this study, we aimed to evaluate serum AMH levels in adolescent and young adult (AYA) Turkish patients with PCOS.

## MATERIALS AND METHODS

Ninety AYA patients with PCOS were recruited consecutively from the adolescent outpatient clinic of Zekai Tahir Burak Women’s Health Education and Research Hospital, between March and June 2014. The diagnosis of PCOS was made in accordance with the recent Amsterdam ESHRE/ASRM proposal, that all three of the Rotterdam criteria were present; polycystic ovaries on ultrasound, menstrual irregularity (chronic anovulation or oligoamenorrhea), and evidence of clinical or biochemical hyperandrogenism criteria as proposed by The National Institutes of Health consensus in 2012^(8)^. Ultrasonographic examinations were performed with using a 3.5 MHz transabdominal convex transducer (SSD 1000; Aloka, Tokyo, Japan) by a single physician. Ovaries were considered polycystic on ultrasound if there were 12 or more follicles measuring 2-9 mm in diameter in each ovary and/or enlarged ovarian volume (>10 mm^3^). Oligomenorrhea was considered as menstrual cycles longer than 45 days, whereas amenorrhea was defined as the absence of a menstrual period for three consecutive months. Chronic anovulation was confirmed with serum levels of progesteron <5 ng/mL during the mid-luteal phase. The modified Ferriman-Gallwey score (FGS), which assesses all 9 body areas, was used to determine the severity of hirsutism^(9)^. Patients with FGS scores ≥8 were considered as having clinical hirsutism. Biochemical hyperandrogenemia was defined as free testosterone (fT) level ≥3.6 ng/mL and/or dehydroepiandrosteronsulfate (DHEA-S) level ≥358 µg/mL^(10)^.

All patients’ disease had been recently diagnosed and therefore they were not yet using oral contraceptives or any other hormonal medications. Exclusion criteria included infectious diseases, use of medications known to alter insulin secretion or action and lipoprotein metabolism, hypertension, smoking, and endocrinopathies including diabetes, Cushing syndrome, androgen-secreting tumors, late-onset 21-hydroxylase deficiency, thyroid dysfunction, hyperprolactinemia, and autoimmune diseases. Forty-seven age-matched healthy AYA patients were also recruited as the control group. The institutional review board approved the study, and written informed consent was obtained from all participants as well as their parents.

A complete physical and pelvic examination was performed in all patients. Gynecologic and general histories were obtained, and demographic characteristics were recorded for each patient. The main parameters recorded were; age, Body mass index (BMI), waist (WC) and hip circumference (HC), age at menarche, menstrual pattern, educational level, socioeconomic status, FGSs, baseline hormone levels, markers of glucose metabolism, serum lipid profile, and AMH levels. The patients’ height and weight were measured using a professional calibrated device. BMI was calculated using the formula BMI=weight (kg)/height (m)^2^. WC was measured at the umbilicus, using a cloth tape, and HC was measured by recording the widest part of the hips.

Antecubital venous blood samples (~10 mL) were obtained from each participant on the second or third day of the menstrual cycle in the morning, following at least an 8-h fasting period, and taken to the laboratory within 10 min. All blood tests except AMH were studied immediately. For AMH, serum samples were separated by centrifugation at 5000 rpm (2236 g) for 10 min., and were stored at -80 °C until use. AMH measurements in the stored serum were performed using a Chromate-4300 plate reader (Awareness Tec. Inc., Palm city, FL, USA) and a commercially available enzyme-linked immunosorbent assay kit (CK-E11351) developed for AMH (Eastbiopharm Co. Ltd., Hangzhou, PRC). The AMH concentration range was determined as 0.01-30 ng/mL, and the minimum detectable dose of this kit was 0.01 ng/mL.

Serum concentrations of baseline hormones and insulin were determined using a UniCel DxI 800 Immunoassay System (Beckman Coulter, Fullerton, CA, USA). Insulin resistance was determined using the homeostasis model assessment (HOMA) with the formula: (fasting serum insulin (mU/mL) x fasting plasma glucose (mmol/L)/22.5). Insulin resistance was defined when values were above 2.5^(11)^. Serum DHEA-S, 17-hydroxy progesterone (17-OH-P), and fT levels were measured using a radioimmunoassay. Serum lipid profile and glucose levels were analyzed using an AU680 Chemistry System (Beckman Coulter, Fullerton, CA, USA).

### Statistical analysis

Data analysis was performed using SPSS for Windows, version 17.0 (SPSS Inc., Chicago, IL, United States). The Kolmogorov-smirnov test was used to test whether continuous variables were normally distributed. Continuous variables were shown as mean ± standard deviation (SD) or median ± interquartile range (IQR), where applicable. Categorical variables were expressed as number (percentage). Mean differences between case and control groups were compared using Student’s t test; the Mann-whitney U test was used for comparisons of the median values. Differences between categorical data were evaluated using the Chi-square test. The optimal cut-off point of the AMH that discriminated groups from each other were evaluated using receiver operating characteristic (ROC) analysis, calculating area under the curve (AUC) as giving the maximum Youden index (sensitivity+specificity-1). P<0.05 was considered to indicate significance.

## RESULTS

A total of 90 consecutive AYA patients (43 with PCOS and 47 age-matched healthy controls) who presented to the adolescent outpatient clinic of our hospital were enrolled in this case-control study. The ages of patients ranged from 15 to 23 years. All study participants had been menstruating for at least three years. Although not statistically significant, median menarch age was 13 years in both groups (range, 11-17 years). The most common presenting symptoms were menstrual irregularity and hirsutism in the study group, whereas it was vaginal discharge in the control group. A significant difference was observed between the two groups in terms of anthropometric measures (p<0.05). The demographic, clinical, and laboratory characteristics of the patients are depicted in Table 1. The mean FGS was 11.1±5.7 in the study group and 5.3±3.6 in the control group; the difference was statistically significant (p<0.001).

Among the markers of glucose metabolism, plasma fasting insulin (p=0.002) and HOMA index (p=0.004) were significantly higher in the PCOS group. The means of LH, and LH to FSH ratio (LH/FSH), DHEA-S, 17-OH-P, and fT were also significantly higher in patients with PCOS. No significant difference was found between the groups regarding the other hormone levels. Lipid profiles were also similar between the two groups (p>0.05).

AMH levels were measurable in all patients. Serum AMH levels were higher in the PCOS group compared with the controls, but the difference was not statistically significant (10.1±6.9 ng/mL vs. 9.4±5.5 ng/mL, p=0.198). There was a significant age-related decrease in AMH levels in both the study and control groups (r=-0.331, p=0.001). There was also a significant inverse correlation between serum AMH and FSH levels in all patients (r=-0.227, p=0.031).

**Figure 1:**
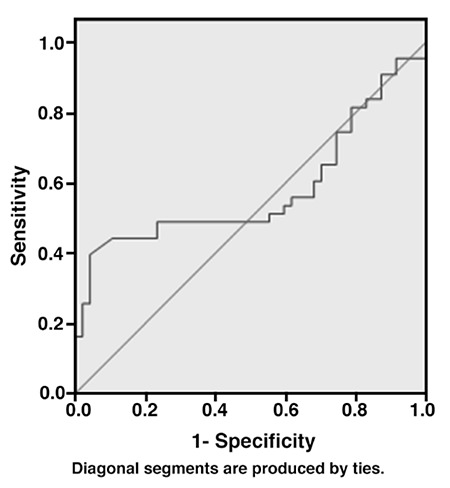
Receiver operator characteristics curve analysis of anti-mullerian hormone to discriminate Polycystic ovary syndrome cases

ROC analyses demonstrated that the AUC indicative of AMH value for discriminating PCOS was 0.579 with a confidence interval of 0.453-0.705. However, the AUC of AMH was not useful to distinguish PCOS (p=0.198). Nevertheless, the cut-off value according to the highest Youden index was calculated to be 14.0 with a sensitivity of 48.8% and specificity of 77.1% (Figure 1).

## DISCUSSION

According to this study, serum AMH levels are slightly higher in AYA Turkish patients than in controls. However, we found a higher cut-off value (14 ng/mL) with a low sensitivity to discriminate patients with PCOS and controls. In addition, AMH was not found to be a good marker for the diagnosis of PCOS in AYA patients.

AMH is a member of the TGF-β family, and is produced by granulosa cells of antral and preantral follicles^(12)^. The main physiologic role of AMH in the ovary seems to be limited to the inhibition of the early stages of follicular development^(13)^ and preventing recruitment of non-dominant follicles^(7)^. Fanchin et al.^(14)^ demonstrated that the antral follicle count (AFC) was closely related to serum AMH levels on cycle day 3 in infertile women. The authors also revealed that the relationship between antral follicle count and AMH was stronger than other hormonal markers. Thus, serum AMH levels are considered to reflect the number of small growing follicles and levels are reduced through reproductive life. Currently, serum AMH levels are often measured during initial laboratory tests for fertility. An age-related decrease in AMH levels and the AFC has previously been reported^(15)^. Age-related reference values for AMH levels were pooling, but reference values have not yet been produced precisely for adolescents. Few studies have evaluated the relationship between AMH and adolescent PCOS.

Several mechanisms have been suggested for the relationship between AMH and PCOS. It was shown that the AFC increased in patients with PCOS, which characterizes the syndrome, and AMH levels were found to correlate with the AFC^(16)^. However, AMH concentration is largely due to the increase in production of AMH by each follicle and not just a consequence of an increase in follicle number. Anovulation,^(17,18)^ hyperandrogenism,^(19)^ and hyperinsulinemia^(20)^ were also suggested to cause elevated AMH levels in patients with PCOS. Nevertheless, the specific underlying mechanism of PCOS needs to be clarified.

Hart et al.^(21)^ evaluated the relationship between AMH, PCO morphology, and PCOS in their large adolescent population. The authors concluded that AMH was not a reliable predictor of PCO morphology and PCOS. A threshold value of 30 pmol/L (4.3 ng/mL) missed 48% cases with PCOS. Li et al.^(22)^ found higher serum AMH levels in adolescent and young adult patients with PCOS than in controls (9.9±4.9 ng/mL vs. 7.1±3.0 ng/mL, p=0.002). The cut-off value for AMH was found to be 8 ng/mL with a specificity of 70% and a sensitivity of 61.7% in that study. Sopher et al.^(23)^ designed a study to determine the diagnostic potential of AMH in non-obese adolescents with PCOS in their small-sized sample. Similar to previous studies, they found that serum AMH levels were higher in patients with PCOS (4.4±3.4 ng/mL vs. 2.4±1.3, ng/mL, p<0.05). They also found higher ovarian and adrenal androgens in patients in PCOS than in controls. The common features of the previous studies were the low sensitivity of AMH and the high specificity for the diagnosis of PCOS. The sensitivity and specificity of AMH in our study was similar to previous studies. We also found higher serum AMH levels in patients with PCOS than in controls; however, this was not statistically significant. Additionally, we found a higher threshold than that reported in the literature. This variation may depend on the diagnostic criteria used and the population studied.

Köninger et al.^(24)^ asserted that there was a relationship between the severity of PCOS and serum AMH levels. The authors suggested that in adolescents for whom vaginal scans are not feasible or in patients without hyperandrogenemia, AMH may be used as a surrogate parameter in the diagnosis of PCOS, superior to androgens and gonadotropins. The severity of PCOS was defined by these authors in accordance with the Rotterdam criteria. However, although all of our patients met all three Rotterdam criteria for PCOS (severe PCOS), AMH was not found as a significant diagnostic marker.

The strength of our study is in its prospective nature. All Rotterdam criteria required for the PCOS diagnosis were present, and there were no interobserver variation; the diagnosis was established by a single physician. The study and control groups were matched in terms of age and BMI. However, we found AMH ineffective at distinguishing AYA patients with PCOS. This may have resulted from the assessment method because serum AMH concentrations may vary depending on the assay method used^(25)^. It may also have been influenced by ethnicity.

In conclusion, we think that AMH is not a good marker for the diagnosis of PCOS in AYA patients. Further randomized controlled studies with more participants are needed to evaluate the diagnostic value of AMH in AYA patients with PCOS.

## Figures and Tables

**Table 1:**
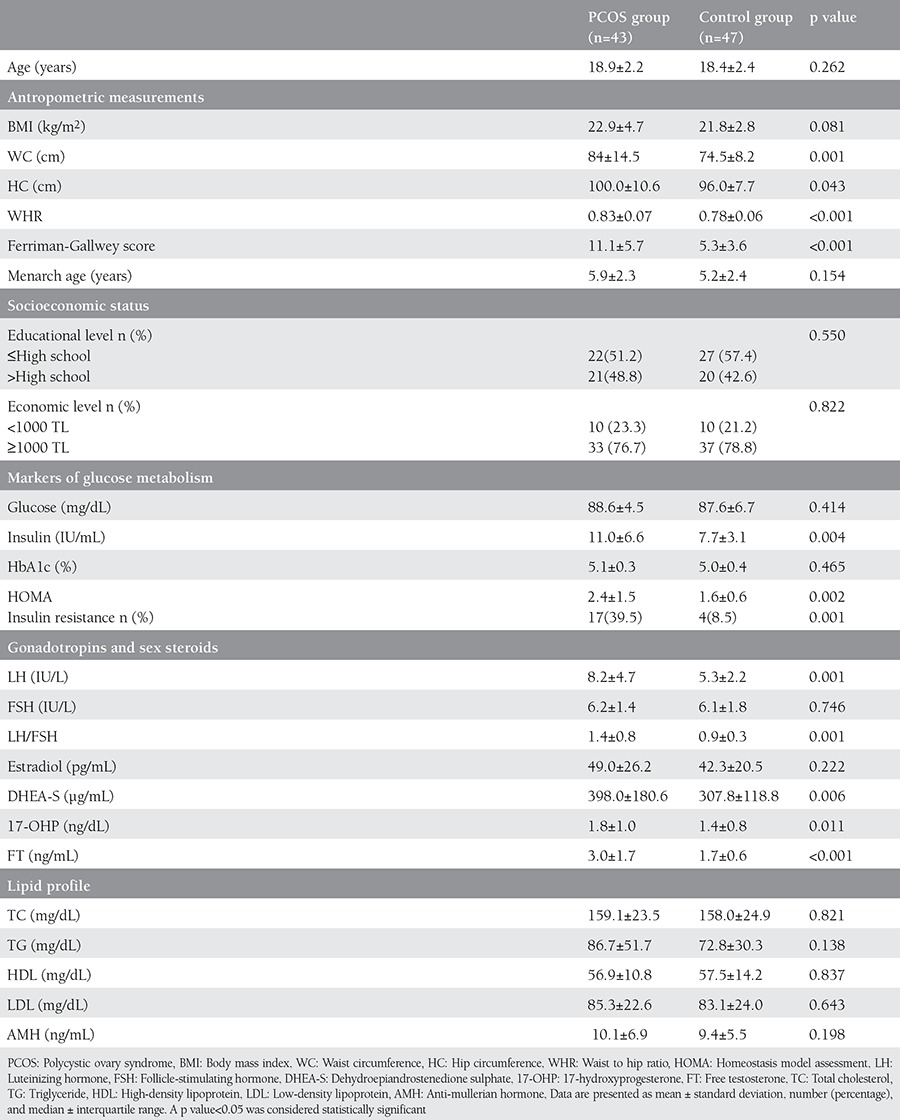
The demographic, clinical, and laboratory characteristics of the patients
